# Artificial Intelligence-Enabled Pharmacovigilance for Neuropsychiatric Therapies: Advancing U.S. Drug Safety Surveillance and Regulatory Decision-Making

**DOI:** 10.7759/cureus.117858

**Published:** 2026-10-13

**Authors:** Rebecca A Omachonu, Sandra A Uzuh, Kehinde Akinola, Kelvin Ovabor, Augustine Odibo

**Affiliations:** 1 Applied Life Sciences, Keck Graduate Institute of Applied Life Sciences, Claremont, USA; 2 Information Science, University at Albany, State University of New York, Albany, USA; 3 Data Science, East Carolina University, Greenville, USA; 4 Information Systems and Analytics, Northern Kentucky University, Highland Heights, USA; 5 Tissue and Biomaterials Engineering, Villanova University, Villanova, USA

**Keywords:** adverse drug reactions, artificial intelligence, drug safety, machine learning, natural language processing, neuropsychiatric therapies, pharmacovigilance, regulatory science, safety surveillance, signal detection

## Abstract

Neuropsychiatric medications, including antidepressants, antipsychotics, mood stabilizers, anxiolytics, and newer agents such as esketamine, are widely prescribed, but their adverse effects are often subjective, delayed in onset, and difficult to separate from the illness being treated. Post-marketing surveillance in the United States relies on the FDA Adverse Event Reporting System (FAERS), MedWatch, the Sentinel System, and Risk Evaluation and Mitigation Strategies (REMS).

This narrative review, reported according to the Scale for the Assessment of Narrative Review Articles (SANRA), examines how well these systems detect neuropsychiatric adverse events and what artificial intelligence (AI) can add. PubMed/MEDLINE, Embase, and Web of Science were searched for literature published since January 2000, supplemented by regulatory and institutional sources.

Spontaneous reporting misses most adverse drug reactions, while stigma, diagnostic overshadowing, and uncertainty about whether a symptom reflects the drug or the disorder further reduce reporting of psychiatric effects. Disproportionality signals, such as those reported for dissociation and suicidal ideation with esketamine, need cautious interpretation because notoriety bias, solicited reporting under REMS, and confounding by indication can inflate them.

Long-latency effects such as tardive dyskinesia and antipsychotic-induced metabolic change fall outside the observation windows of premarket trials and most spontaneous reports. Natural language processing (NLP) can retrieve adverse events documented in free-text psychiatric records with good accuracy, and large language models (LLMs) show early promise for structuring adverse event narratives, but most AI studies are retrospective proofs of concept, and models trained on biased data can reproduce existing inequities.

AI is therefore best used to widen and prioritize signal detection, with causality assessment and regulatory decisions remaining with clinicians and regulators. Priorities include prospective evaluation of AI tools in live pharmacovigilance workflows, linkage of FAERS with electronic health record and claims data, point-of-care reporting tools, and efforts to reduce the stigma that discourages reporting of psychiatric adverse effects.

## Introduction and background

Mental illness carries a large public health burden in the United States. In the 2021 National Survey on Drug Use and Health, 21.0 million U.S. adults (8.3%) reported a major depressive episode in the past year [1]. The lifetime morbid risk of major depression has been estimated at 29.9% [2], and a meta-analysis of 33 studies of veterans of the wars in Iraq and Afghanistan estimated the prevalence of post-traumatic stress disorder (PTSD) at 23% [3]. The incremental economic burden of adults with major depressive disorder alone was estimated at $326.2 billion in 2018 (in 2020 U.S. dollars), 37.9% higher than in 2010 [4]. Because psychotropic drugs are taken by many people, often for years, even small differences in their safety have large consequences at the population level.

Treatment resistance has driven the development of mechanistically novel agents, including the N-methyl-D-aspartate (NMDA) receptor modulators ketamine and esketamine [5], and renewed trials of psychedelics such as psilocybin [6]. These agents bring risks that were less prominent with older drugs, including dissociation, sedation, abuse liability, blood-pressure increases, and uncertain long-term effects, which premarket trials of limited size and duration cannot fully characterize.

Pharmacovigilance is defined by the World Health Organization as the science and activities relating to the detection, assessment, understanding, and prevention of adverse effects or any other medicine-related problem [7]. Three terms recur in this review. A safety signal is information suggesting a new, potentially causal association between a drug and an adverse event, or a new aspect of a known association, that warrants further evaluation [7]. A boxed (“black-box”) warning is the most prominent warning the U.S. Food and Drug Administration (FDA) can require in prescription drug labeling. Passive surveillance depends on clinicians, patients, and manufacturers submitting reports of suspected adverse events, whereas active surveillance searches existing healthcare data, such as insurance claims and electronic health records (EHRs), for adverse outcomes without waiting for reports.

In the United States, the FDA coordinates post-marketing safety monitoring [8]. The FDA Adverse Event Reporting System (FAERS) is the national database of spontaneous reports, which clinicians and patients can submit directly through MedWatch and which manufacturers are required to forward [9]. The Sentinel System is the FDA’s active surveillance system, querying claims and EHR data held by partner organizations [10,11]. When signals emerge, the FDA can require labeling changes and post-marketing studies [12], and for selected high-risk drugs it can require a Risk Evaluation and Mitigation Strategy (REMS) with additional safeguards [13]. Esketamine nasal spray, for example, is available only through a REMS that restricts its use to certified healthcare settings because of the risks of sedation, dissociation, and abuse and misuse [14,15]. Clozapine was also managed under a REMS for severe neutropenia until the FDA removed that program in June 2025; neutrophil monitoring is now addressed through labeling [16].

These systems face particular difficulties with neuropsychiatric drugs. Many adverse effects are subjective changes in mood, cognition, sleep, or behavior that overlap with symptoms of the disorder being treated [17,18]. Spontaneous reporting also misses most adverse drug reactions in every therapeutic area: a systematic review of 37 studies from 12 countries found a median underreporting rate of 94% (interquartile range (IQR) 82%-98%) [19]. That estimate is not specific to psychiatric adverse events, and no comparable pooled estimate exists for them. The risk of suicidality in antidepressant-treated youth shows how hard these signals can be to establish: it was confirmed through pooled analysis of placebo-controlled trials rather than spontaneous reports, and led in October 2004 to an FDA directive to add a boxed warning to all antidepressants [20,21].

Machine learning, natural language processing (NLP), and large language models (LLMs) can analyze large linked datasets and unstructured clinical text that manual review cannot cover [22-24]. The objective of this review is to examine (i) how well existing U.S. pharmacovigilance systems detect neuropsychiatric adverse events, (ii) the features of this drug class that make detection difficult, and (iii) the evidence on AI methods for closing these gaps and their limitations, and to identify implications for regulators, manufacturers, and health systems.

## Review

Methods

Design

This is a narrative review. A narrative design was chosen over a systematic review because the question spans pharmacovigilance, psychiatry, artificial intelligence (AI), health informatics, and regulatory science, whose sources (empirical studies, reviews, regulatory documents, and policy reports) use different designs and outcomes that cannot be pooled. The review is reported according to the Scale for the Assessment of Narrative Review Articles (SANRA), which covers justification of the topic, statement of aims, description of the literature search, referencing, scientific reasoning, and presentation of relevant data [25], and follows published guidance on writing narrative reviews [26]. It does not claim the reproducibility or formal bias appraisal of a systematic review.

Search Strategy

PubMed/MEDLINE, Embase, and Web of Science were searched for records published from January 1, 2000, to June 30, 2026. Search terms were organized into four concept domains and combined as Domain 1 AND Domain 2 AND (Domain 3 OR Domain 4) (Table 1); the strings for each database, including controlled-vocabulary (MeSH and Emtree) mapping and field tags, are given in Appendix 1. The database searches were used to map the literature rather than to compile an exhaustive set of records. Most sources were identified through targeted searching for specific topics, the reference lists of key reviews, and the authors’ knowledge of the field. Grey literature was identified from the websites of the FDA, the World Health Organization, and the Substance Abuse and Mental Health Services Administration, using the site-restricted searches listed in Appendix 1. During revision in October 2026, a supplementary targeted search for signal-detection methodology and its biases, psychiatric-specific NLP, primary FDA documents on REMS and boxed warnings, and regulatory guidance on AI identified 20 additional sources.

**Table 1:** Search strategy, information sources, and concept domains used to identify literature on AI-enabled pharmacovigilance of neuropsychiatric therapies

Search component	Search strategy
Search period	January 1, 2000, to June 30, 2026
Databases	PubMed/MEDLINE, Embase, and Web of Science (exact strings in Appendix Table S1)
Grey literature	FDA guidance documents, Drug Safety Communications, REMS documents, FAERS resources, and Sentinel System assessments; World Health Organization pharmacovigilance publications; SAMHSA national survey reports
Other methods	Targeted topic searches; reference lists of key reviews; supplementary targeted search during revision (October 2026)
Domain 1: Pharmacovigilance and drug safety	"pharmacovigilance" OR "drug safety surveillance" OR "post-marketing surveillance" OR "adverse drug event*" OR "adverse drug reaction*" OR "signal detection" OR "disproportionality analysis" OR "under-reporting"
Domain 2: Neuropsychiatric therapies	"neuropsychiatric" OR "psychotropic" OR "antidepressant*" OR "antipsychotic*" OR "mood stabilizer*" OR "anxiolytic*" OR "ketamine" OR "esketamine" OR "psilocybin" OR "psychotropic polypharmacy" OR "tardive dyskinesia" OR "suicidality"
Domain 3: Artificial intelligence and data-driven methods	"artificial intelligence" OR "machine learning" OR "deep learning" OR "natural language processing" OR "large language model*" OR "text mining" OR "digital phenotyping" OR "algorithmic bias"
Domain 4: U.S. regulatory systems and real-world data	"FAERS" OR "FDA Adverse Event Reporting System" OR "MedWatch" OR "Sentinel Initiative" OR "Risk Evaluation and Mitigation Strateg*" OR "REMS" OR "electronic health record*" OR "claims data" OR "real-world evidence"
Boolean combination	Terms within each domain combined with OR; domains combined as Domain 1 AND Domain 2 AND (Domain 3 OR Domain 4)
Limits	English language; publication date January 1, 2000, onward (earlier foundational sources retained where essential)

Eligibility and Selection

Sources were selected purposively by the authors for their relevance to the review questions, using the criteria in Table 2 (relevance, therapeutic scope, study type, language, setting, methodological adequacy, and availability) as a guide; uncertainties were resolved by discussion among the authors. Journal impact and citation counts were not used as selection criteria. Because selection was purposive rather than exhaustive, the number of records at each screening stage is not reported, and the review does not claim the completeness of a systematic review. All 75 included sources are listed with their design and thematic domain in Appendix 2.

**Table 2:** Eligibility criteria for source selection

Criterion	Inclusion criteria	Exclusion criteria
Publication period	Published January 2000 onward; earlier foundational sources retained where essential (e.g., WHO pharmacovigilance definition)	Sources superseded by more recent evidence
Relevance to pharmacovigilance	Addresses adverse event detection, reporting, signal detection, underreporting, or post-marketing safety surveillance	No connection to drug safety monitoring or adverse event assessment
Neuropsychiatric therapeutics	Concerns antidepressants, antipsychotics, mood stabilizers, anxiolytics, or novel agents (ketamine, esketamine, psychedelics), or neuropsychiatric adverse effects	Non-psychotropic drug classes with no transferable relevance to neuropsychiatric safety
AI and data-driven approaches	Examines machine learning, natural language processing (NLP), large language models (LLMs), longitudinal modeling, digital phenotyping, or multi-source data integration for drug safety	AI used only for purposes unrelated to safety surveillance or clinical decision support
Study type	Empirical studies, systematic and scoping reviews, meta-analyses, regulatory guidance, and health-policy reports from recognized bodies	Opinion pieces, editorials, and commentaries without substantive evidence; unverifiable sources
Language	English	Other languages
Research setting	U.S. healthcare and regulatory context, or international evidence applicable to U.S. practice	Settings with no transferability to U.S. clinical or regulatory practice
Methodological adequacy	Methods, data sources, and limitations clearly described	Insufficient methodological detail or unclear data provenance
Availability	Full text accessible	Abstract only, or full text unobtainable

Data Extraction and Synthesis

Information from each source was assigned to one or more of five domains: (i) structure and limitations of existing pharmacovigilance systems; (ii) neuropsychiatric-specific safety challenges; (iii) AI techniques and applications in drug safety; (iv) evidence of AI effectiveness and limitations; and (v) regulatory, ethical, and equity considerations. Findings were synthesized narratively. Where sources disagreed, the disagreement is reported rather than resolved, and more weight was given to designs better suited to the question (systematic reviews and meta-analyses, then large pharmacoepidemiological studies, then single-site studies and expert commentary); regulatory documents were used to describe rules and programs, not to estimate effects. Quantitative figures are reported as findings of the cited study, with the original estimate, denominator, and interval where available. The conceptual framework (Figure 1) was derived by grouping the extracted themes: Domains 1 and 2 describe the gaps, Domains 3 and 4 the analytic capabilities that address them, and Domain 5 the conditions for acting on their outputs.

Quality Appraisal

No formal risk-of-bias instrument (such as AMSTAR-2 or ROBIS) was applied, because these tools are designed for systematic reviews of homogeneous study types. The design of each source is recorded in Appendix 2 and was considered when weighting findings. This is a limitation of the review. The review used only published literature and publicly available documents, so ethical approval was not required.

Results

Underreporting and Its Drivers

Hazell and Shakir’s median underreporting estimate of 94% (IQR 82%-98%) spans all therapeutic areas [19]; the figure often quoted from it, that only 1%-10% of reactions are reported, should therefore not be read as a psychiatric estimate. Underreporting coexists with large and rising report volumes: serious adverse drug events reported to the FDA rose 2.6-fold between 1998 and 2005 [27]. The problem is therefore less the number of reports than which events reach the system. Among health professionals, the main determinants of underreporting include not knowing what or how to report, reluctance to report a reaction unless certain the drug caused it, the belief that serious reactions are already well documented, and lack of time [28].

Several features of psychiatric care add to these general barriers. Stigma deters people with mental illness from seeking and staying in care [29], and experienced and anticipated discrimination is common among people with schizophrenia across countries [30]; both reduce the contacts at which adverse effects might be recognized. Some adverse effects, notably sexual dysfunction, are rarely volunteered by patients or asked about by clinicians, so they go unrecorded unless specifically elicited [31]. Diagnostic overshadowing, in which new symptoms in a person with known mental illness are attributed to that illness, has been documented in emergency departments [32] and can divert attention from possible drug effects. These mechanisms are well described qualitatively, but we found no study that measured their effect on reporting rates for psychiatric adverse events.

Interpreting Spontaneous-Report Signals

FAERS is analyzed mainly by disproportionality analysis, which compares how often an event is reported with a drug relative to all other drugs in the database [33]. Common measures are the proportional reporting ratio, the reporting odds ratio (ROR), the information component, and the empirical Bayes geometric mean (EBGM); the Bayesian measures shrink estimates based on few reports toward the null to reduce false positives [34,35]. A retrospective evaluation in the European database suggested that statistical screening could have identified some signals earlier than routine review [36]. These measures describe reporting patterns, not incidence, and cannot establish causation. They are also distorted by factors that change reporting independently of risk. Media attention or regulatory alerts increase reporting of a specific drug-event pair (notoriety bias) [37]; programs that solicit reports, including some REMS, add reports that would otherwise not be submitted (stimulated reporting); and the classical Weber effect, a peak in reporting about two years after launch, does not reliably hold in modern FAERS, where reports for most drugs approved in 2006-2010 rose over the first three quarters and then remained roughly constant [38].

Esketamine illustrates these issues. Gastaldon et al. analyzed 962 esketamine cases reported to FAERS in the drug’s first year on the market (March 2019 to March 2020) [14]. They found disproportionality signals for dissociation (ROR 1,612.64; 95% CI 1,354.63-1,919.79), sedation (ROR 238.46; 95% CI 202.98-280.15), feeling drunk (ROR 96.17; 95% CI 61.42-150.57), suicidal ideation (ROR 24.03; 95% CI 18.72-30.84), and completed suicide (ROR 5.75; 95% CI 3.18-10.41). When esketamine was compared with venlafaxine rather than with all drugs, the signal for suicidal and self-injurious ideation persisted, but those for suicide attempt and completed suicide did not. Three factors complicate interpretation. The REMS solicits reports of sedation and dissociation, which inflates their apparent disproportionality; patients receiving esketamine have treatment-resistant depression and a high baseline risk of suicidal ideation (confounding by indication), which comparison with all FAERS drugs does not address; and publicity around a novel agent may increase reporting. The analysis was therefore hypothesis-generating rather than evidence of excess risk, and it shows why statistical signals, whether produced by conventional or AI-based methods, need clinical review before action.

Subjective, Overlapping, and Long-Latency Adverse Effects

Many neuropsychiatric adverse effects, such as changes in mood, motivation, cognition, sleep, and emotional range, are known mainly through the patient’s own account and have no laboratory or imaging marker [17]. Data from routine practice show that sexual dysfunction, drowsiness, and weight gain are among the effects most often reported by people taking selective serotonin reuptake inhibitors [18]. Patient-rated instruments such as the Glasgow Antipsychotic Side-Effect Scale can make these effects visible in routine care [39], but they are not used systematically, and their results rarely reach surveillance databases.

Separating a drug effect from the illness is hardest for suicidality, anxiety, agitation, and insomnia, which are both symptoms of depression and possible adverse effects of treatment. The FDA’s pooled analysis of 24 placebo-controlled pediatric trials, involving more than 4,400 patients, found suicidal thinking or behavior in 4% of patients receiving antidepressants and 2% receiving placebo, with no completed suicides [20]; across all drugs and indications, the relative risk was 1.95 (95% CI 1.28-2.98) [21]. Because suicidality also occurs in untreated depression, this risk could be identified only against a randomized placebo group; spontaneous reports alone could not separate it from the background rate. A later analysis of adult trials found that the risk varied with age, being raised in those under 25 years and lower in older adults [40], and the warning was subsequently extended to young adults [41].

Some of the most burdensome effects emerge only after months or years. A meta-analysis of 41 studies (11,493 participants) estimated the prevalence of tardive dyskinesia at 25.3% (95% CI 22.7-28.1%) among people treated with antipsychotics: 20.7% with current second-generation and 30.0% with current first-generation agents [42]. Antipsychotics also cause weight gain, dyslipidemia, hyperglycemia, and metabolic syndrome, which raise cardiovascular risk [43]. A 2004 consensus statement recommended baseline and follow-up monitoring of weight, glucose, and lipids [44], yet such monitoring remains inconsistent [43]. Tardive dyskinesia had no approved treatment until the vesicular monoamine transporter 2 (VMAT2) inhibitors valbenazine and deutetrabenazine were approved in 2017 [45]. Premarket trials, which usually last weeks to months, cannot observe these outcomes, and patients and clinicians are unlikely to link a gradually emerging problem to a drug started years earlier, so spontaneous reporting captures them poorly as well.

Polypharmacy compounds these problems. In U.S. office-based psychiatry, the proportion of visits at which three or more psychotropic drugs were prescribed rose from 16.9% in 1996-1997 to 33.2% in 2005-2006 [46], and polypharmacy is most common in severe and treatment-resistant illness [47]. Definitions of polypharmacy range from two to five or more concurrent drugs [48], which complicates comparison across studies. When several drugs are started or adjusted together, a report can only name suspected agents, and drug-drug interactions are difficult to detect from individual reports.

Limits of Premarket Evidence

Premarket trials exclude many of the patients who later receive these drugs. Most patients treated for depression in a routine outpatient practice would not have qualified for typical antidepressant efficacy trials [49], and reviews across therapeutic areas find that trial samples commonly differ from treated populations in age, comorbidity, and severity [50]. In trials of four widely prescribed drugs, the proportion of participants aged 65 years or older was less than half that in the treated population in 38%-70% of trial reports [51], and pregnant and lactating individuals are usually excluded from drug development altogether [52]. Of 222 novel therapeutics approved by the FDA in 2001-2010, 71 (32.0%) later had a post-marketing safety event (withdrawal, boxed warning, or safety communication), at a median of 4.2 years after approval, and psychiatric therapeutics were among the categories with significantly higher rates [53].

Active Surveillance and Data Integration

Sentinel complements FAERS by running standardized queries across distributed claims and EHR data, with partner organizations keeping their data locally and returning aggregate results [10,54]. It now supports regulatory decisions routinely, including through its Active Risk Identification and Analysis (ARIA) system [11]. Its advantages for neuropsychiatric safety are exposure denominators and longer follow-up, which spontaneous reports lack. Its main weakness for this field is that claims data record diagnoses and dispensing but few symptoms, so outcomes such as suicidal ideation, sexual dysfunction, or cognitive change are poorly captured [54]. The FDA’s most recent assessment of Sentinel, covering 2022 to 2024, identifies integration of EHR data within a causal-analysis framework as a priority [55]. The literature differs on whether these improvements will be enough: some authors regard Sentinel’s longitudinal follow-up as a substantial advance for delayed outcomes [11], whereas others emphasize fragmentation across data partners, inconsistent outcome definitions, and limited power for rare events [54].

Other data streams could fill some gaps. Posts on social media and health forums contain first-person descriptions of adverse effects, and NLP methods can extract them [56-58], although these data are unverified, unrepresentative, and lack exposure denominators. Linking FAERS, EHR, claims, and patient-generated data is limited by privacy law, inconsistent data standards, and variable data quality. Privacy-preserving methods such as federated learning, in which models are trained across institutions without pooling raw records, may reduce some of these barriers [59].

AI Methods and Their Evidence

Machine learning extends disproportionality analysis to multivariable models that can adjust for co-medication and other confounders in spontaneous-report and observational data [22]. NLP has long been used to extract adverse drug events from clinical notes, the biomedical literature, and social media [23], and a 2025 scoping review mapped a rapidly growing body of studies applying NLP and machine learning to adverse event detection in EHRs [24]. The most directly relevant psychiatric example is ADEPt, a pipeline applied to de-identified records of about 264,000 patients from four UK psychiatric hospitals. It identified 19 adverse effects of antipsychotics and antidepressants and distinguished affirmed from hypothetical or historical mentions, with an average F-measure and accuracy of 0.83 [60]. This shows that adverse events documented narratively in psychiatric notes can be retrieved at scale, although performance was measured retrospectively within one health system.

Since about 2023, generative LLMs have been tested for extracting structured adverse event information from free text, for example from influenza vaccine adverse event reports [61] and in benchmark pharmacovigilance event-extraction tasks, where ChatGPT performed reasonably but below fine-tuned models [62]. A 2025 review concluded that LLMs are promising for adverse event detection and summarization, but that hallucination, limited interpretability, and the absence of prospective validation remain unresolved [63]. A 2024 scoping review of AI applied to structured real-world data similarly found that most studies were proofs of concept, with few evaluated in live pharmacovigilance workflows [64].

Deep learning models of sequential EHR data, such as RETAIN and Deep Patient, represent a patient’s history over time and predict future diagnoses [65,66]. They were not designed for pharmacovigilance, but the same approach could be used to look for gradually emerging outcomes such as tardive dyskinesia or metabolic change after drug exposure; we found no published application of this kind in psychiatry. Digital phenotyping, the continuous measurement of behavior from smartphone and wearable sensors such as activity, sleep, and phone use, has been developed to measure psychiatric symptoms [67,68]. It is a potential future data source for monitoring drug effects, not yet an established pharmacovigilance tool.

Limits of AI and the Need for Human Oversight

The performance of AI models is bounded by the quality and representativeness of their training data, and their outputs require expert interpretation [69,70]. Obermeyer et al. showed that a widely used commercial algorithm underestimated the health needs of Black patients because it used past healthcare costs as a proxy for need [71], and models trained on data shaped by unequal access to care can reproduce those inequities [72]. Spontaneous reports and psychiatric records reflect who reaches care, who is believed, and who reports, so a model trained on them could under-detect adverse effects in groups that are already under-recognized.

Pharmacovigilance specialists at the FDA have argued that current AI is useful for case processing, such as triaging reports and coding narratives, but that its outputs need human oversight and quality assurance before they inform regulatory decisions [73]. Adoption has also lagged behind technical capability because of validation, governance, and workflow barriers [74]. The FDA’s January 2025 draft guidance on AI to support regulatory decision-making, whose scope includes post-marketing uses, proposes a risk-based credibility assessment in which the evidence required for a model depends on its context of use and the consequences of an error [75].

Discussion

Figure 1 summarizes the conceptual framework derived from the review. Existing pharmacovigilance infrastructure connects, through real-time data monitoring, to the neuropsychiatric challenges described above and, through advanced analytics, to AI-enabled methods. Three conditions link these elements to more responsive safety monitoring: improved reporting, integrated data, and human-AI collaboration.

**Figure 1:**
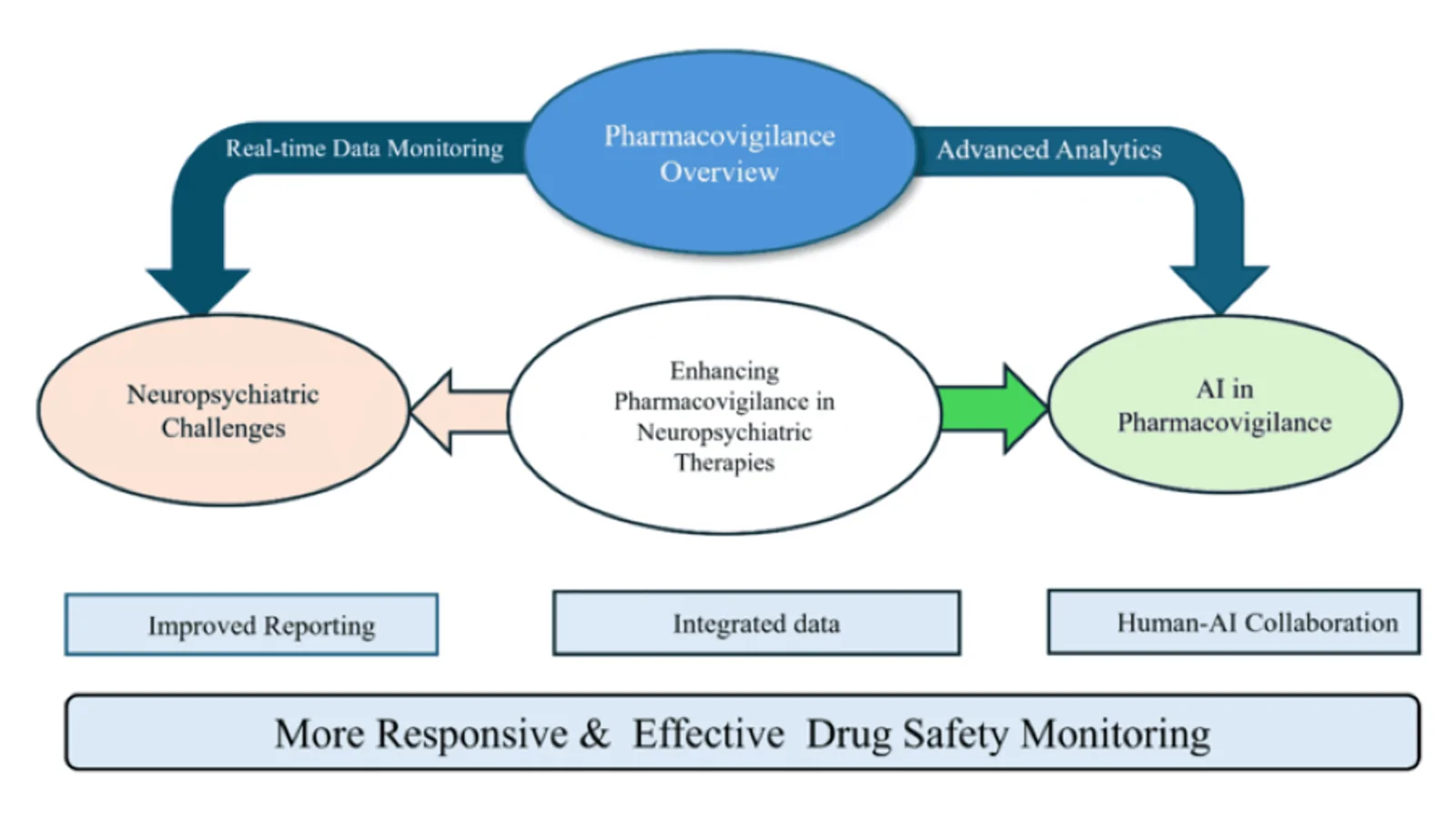
Conceptual framework for enhancing pharmacovigilance in neuropsychiatric therapies

The framework links existing pharmacovigilance infrastructure, through real-time data monitoring and advanced analytics, to neuropsychiatric-specific challenges and AI-enabled methods, and identifies improved reporting, integrated data, and human-AI collaboration as the conditions for more responsive drug safety monitoring. The figure was created by the authors using Microsoft PowerPoint (Microsoft Corporation, Redmond, WA, USA).

The main weakness the review identifies is not a lack of surveillance infrastructure but the selection of events that reach it. Events that are subjective, delayed, stigmatized, or confounded by the illness being treated are the least likely to be reported, and these are the typical features of neuropsychiatric adverse effects. The evidence suggests that each gap calls for a different response. Underreporting and subjectivity are best addressed by retrieving events already documented in clinical notes, where NLP has the strongest psychiatric evidence [60], and by routine use of structured patient-rated measures [39]. Long-latency effects require active surveillance with exposure denominators and long follow-up [11], possibly supported by longitudinal models. Confounding by indication and polypharmacy require active-comparator and multivariable methods rather than raw disproportionality [14,22]. In each case, AI mainly improves the first steps (capturing, structuring, and prioritizing events) and does little for the last step, causal inference, which still depends on study design and clinical judgment.

The literature also disagrees on strategy. Some authors favor incremental improvement through clinician education, simpler reporting, and process refinement [28], whereas others favor integrated multi-source analytics [64,74]. Because almost no AI tools have been evaluated prospectively in live pharmacovigilance workflows [63,64], the evidence does not yet favor one approach, and the two are complementary: better reporting improves the data on which AI depends.

Several practical implications follow. For regulators, priorities are to extend Sentinel’s use of EHR data to psychiatric outcomes that claims data miss [55], to apply the FDA’s credibility framework to AI tools used in signal detection [75], and to interpret psychiatric FAERS signals with explicit attention to notoriety bias, solicited REMS reports, and confounding by indication [14,37]. For manufacturers, priorities are validated and publicly described analytic pipelines, systematic collection of patient-reported outcomes in post-marketing studies, and long-term follow-up for outcomes such as tardive dyskinesia and metabolic change [42,43]. For health systems and clinicians, priorities are low-burden reporting tools embedded in the EHR at the point of care, routine metabolic monitoring of patients taking antipsychotics [44], and specific questioning about sensitive effects such as sexual dysfunction [31], together with efforts to reduce the stigma that discourages patients from disclosing them [29].

This review brings together pharmacovigilance infrastructure, the clinical features of neuropsychiatric drug safety, AI methods, and regulatory science, which are usually reviewed separately. Its main contribution is to distinguish where AI can help (capturing and prioritizing events that current systems miss) from where it cannot (establishing causation and deciding on regulatory action), and to link each documented gap to the type of response the evidence supports.

Limitations

This review has several limitations. As a narrative review with purposive source selection, it is open to selection bias; the database searches were not screened exhaustively, and no formal risk-of-bias instrument was applied. Only English-language sources were included. Much of the AI evidence comes from retrospective, single-institution studies, so performance in live, cross-institutional workflows is largely unknown [63,64]. The best available underreporting estimate is not specific to psychiatric adverse events [19], and the effect of stigma and diagnostic overshadowing on reporting has not been quantified. The regulatory landscape is changing quickly, as shown by the removal of the clozapine REMS in 2025 [16], and AI methods, particularly LLMs, are evolving faster than their evaluation, so developments after the search date may change these conclusions.

Future research should evaluate AI-enabled pharmacovigilance tools prospectively in live settings, including their effect on workflows and decisions; measure underreporting of psychiatric adverse events directly; study real-world safety in populations underrepresented in trials, including older adults and pregnant and lactating individuals [51,52]; and audit AI tools for differential performance across demographic groups [72].

## Conclusions

Current U.S. pharmacovigilance systems provide an essential foundation but systematically miss the adverse effects most typical of neuropsychiatric drugs: subjective, delayed, stigmatized, and confounded by the illness being treated. AI, particularly NLP applied to clinical notes, can widen and prioritize the events that reach reviewers, but current evidence is mostly retrospective, and causality assessment and regulatory decisions should remain with clinicians and regulators. Practical next steps are prospective evaluation of AI tools, linkage of EHR data to active surveillance for psychiatric outcomes, point-of-care reporting, and reduction of the stigma that discourages reporting.

## Appendices

Appendix 1

**Table 3:** Exact search strings run in each database

Database	Search string
PubMed/MEDLINE	#1 "Pharmacovigilance"[Mesh] OR "Adverse Drug Reaction Reporting Systems"[Mesh] OR "Product Surveillance, Postmarketing"[Mesh] OR "Drug-Related Side Effects and Adverse Reactions"[Mesh] OR pharmacovigilance[tiab] OR "drug safety surveillance"[tiab] OR "post-marketing surveillance"[tiab] OR "postmarketing surveillance"[tiab] OR "adverse drug event*"[tiab] OR "adverse drug reaction*"[tiab] OR "signal detection"[tiab] OR "disproportionality analysis"[tiab] OR under-reporting[tiab] OR underreporting[tiab] #2 "Psychotropic Drugs"[Mesh] OR "Antidepressive Agents"[Mesh] OR "Antipsychotic Agents"[Mesh] OR "Anti-Anxiety Agents"[Mesh] OR "Antimanic Agents"[Mesh] OR "Ketamine"[Mesh] OR "Psilocybin"[Mesh] OR "Tardive Dyskinesia"[Mesh] OR "Suicidal Ideation"[Mesh] OR neuropsychiatric[tiab] OR psychotropic*[tiab] OR antidepressant*[tiab] OR antipsychotic*[tiab] OR "mood stabilizer*"[tiab] OR anxiolytic*[tiab] OR ketamine[tiab] OR esketamine[tiab] OR psilocybin[tiab] OR "tardive dyskinesia"[tiab] OR suicidality[tiab] #3 "Artificial Intelligence"[Mesh] OR "Machine Learning"[Mesh] OR "Deep Learning"[Mesh] OR "Natural Language Processing"[Mesh] OR "artificial intelligence"[tiab] OR "machine learning"[tiab] OR "deep learning"[tiab] OR "natural language processing"[tiab] OR "large language model*"[tiab] OR "text mining"[tiab] OR "digital phenotyping"[tiab] OR "algorithmic bias"[tiab] #4 "Electronic Health Records"[Mesh] OR FAERS[tiab] OR "Adverse Event Reporting System"[tiab] OR MedWatch[tiab] OR Sentinel[tiab] OR REMS[tiab] OR "Risk Evaluation and Mitigation Strateg*"[tiab] OR "electronic health record*"[tiab] OR "claims data"[tiab] OR "real-world evidence"[tiab] #5 #1 AND #2 AND (#3 OR #4); filters: English; publication date 2000/01/01 to 2026/06/30 (searched June 30, 2026)
Embase (Embase.com)	#1 ‘pharmacovigilance’/exp OR ‘postmarketing surveillance’/exp OR ‘adverse drug reaction’/exp OR (pharmacovigilance OR ‘drug safety surveillance’ OR ‘post-marketing surveillance’ OR ‘adverse drug event*’ OR ‘adverse drug reaction*’ OR ‘signal detection’ OR ‘disproportionality analysis’ OR underreporting OR ‘under-reporting’):ti,ab #2 ‘psychotropic agent’/exp OR ‘antidepressant agent’/exp OR ‘neuroleptic agent’/exp OR ‘anxiolytic agent’/exp OR ‘mood stabilizer’/exp OR ‘ketamine’/exp OR ‘esketamine’/exp OR ‘psilocybine’/exp OR ‘tardive dyskinesia’/exp OR ‘suicidal ideation’/exp OR (neuropsychiatric OR psychotropic* OR antidepressant* OR antipsychotic* OR ‘mood stabilizer*’ OR anxiolytic* OR ketamine OR esketamine OR psilocybin OR ‘tardive dyskinesia’ OR suicidality):ti,ab #3 ‘artificial intelligence’/exp OR ‘machine learning’/exp OR ‘deep learning’/exp OR ‘natural language processing’/exp OR (‘artificial intelligence’ OR ‘machine learning’ OR ‘deep learning’ OR ‘natural language processing’ OR ‘large language model*’ OR ‘text mining’ OR ‘digital phenotyping’ OR ‘algorithmic bias’):ti,ab #4 ‘electronic health record’/exp OR (faers OR ‘adverse event reporting system’ OR medwatch OR sentinel OR rems OR ‘risk evaluation and mitigation strateg*’ OR ‘electronic health record*’ OR ‘claims data’ OR ‘real-world evidence’):ti,ab #5 #1 AND #2 AND (#3 OR #4) AND [english]/lim Limits: English language; publication years 2000 to 2026.
Web of Science Core Collection	#1 TS=(pharmacovigilance OR "drug safety surveillance" OR "post-marketing surveillance" OR "postmarketing surveillance" OR "adverse drug event*" OR "adverse drug reaction*" OR "signal detection" OR "disproportionality analysis" OR underreporting OR "under-reporting") #2 TS=(neuropsychiatric OR psychotropic* OR antidepressant* OR antipsychotic* OR "mood stabilizer*" OR anxiolytic* OR ketamine OR esketamine OR psilocybin OR "tardive dyskinesia" OR suicidality) #3 TS=("artificial intelligence" OR "machine learning" OR "deep learning" OR "natural language processing" OR "large language model*" OR "text mining" OR "digital phenotyping" OR "algorithmic bias") #4 TS=(FAERS OR "Adverse Event Reporting System" OR MedWatch OR Sentinel OR REMS OR "Risk Evaluation and Mitigation Strateg*" OR "electronic health record*" OR "claims data" OR "real-world evidence") #5 #1 AND #2 AND (#3 OR #4); refined by Languages: English; Publication Years: 2000-2026
Grey literature (site-restricted web searches)	FDA (fda.gov): (1) antidepressant suicidality boxed warning children adolescents; (2) esketamine Spravato REMS; (3) clozapine REMS removal; (4) FDA Adverse Event Reporting System FAERS data; (5) Sentinel System assessment active surveillance; (6) artificial intelligence regulatory decision-making drug biological products guidance; (7) postmarketing requirements risk evaluation mitigation strategies drug safety WHO (who.int): (8) pharmacovigilance safety monitoring of medicinal products SAMHSA (samhsa.gov): (9) National Survey on Drug Use and Health major depressive episode adults key indicators

Appendix 2

Domains: 1, structure and limitations of pharmacovigilance systems; 2, neuropsychiatric-specific safety challenges; 3, AI techniques and applications; 4, evidence of AI effectiveness and limitations; 5, regulatory, ethical, and equity considerations. "Background" and "Methods" denote sources used only for disease burden or review methodology.

**Table 4:** Sources included in the review, with design and thematic domain

No.	Source	Design	Domain	Used for
1	SAMHSA, 2022	National survey report	Background	Prevalence of major depressive episode
2	Kessler et al., 2012	Epidemiological survey analysis	Background	Lifetime morbid risk of major depression
3	Fulton et al., 2015	Meta-analysis	Background	PTSD prevalence in veterans
4	Greenberg et al., 2021	Economic burden study	Background	Economic burden of major depressive disorder
5	Krystal et al., 2019	Narrative review	2	Novel NMDA-modulating antidepressants
6	Carhart-Harris et al., 2021	Randomized controlled trial	2	Psychedelic therapies
7	WHO, 2002	International agency report	1	Definitions of pharmacovigilance and signal
8	FDA, 2024	Regulatory web resource	1	FDA role in post-marketing safety
9	FDA, 2024	Regulatory data resource	1	Description of FAERS
10	Behrman et al., 2011	Perspective (regulatory)	1	Design of Sentinel
11	Platt et al., 2018	Perspective (regulatory)	1	Sentinel use and ARIA
12	FDA, 2019	Regulatory guidance	1, 5	FDA authority for post-marketing requirements
13	FDA, 2024	Regulatory web resource	1, 5	Description of REMS
14	Gastaldon et al., 2021	Disproportionality analysis (FAERS)	1, 2	Esketamine signals
15	FDA, 2026	Regulatory document	1, 5	Current esketamine REMS
16	FDA, 2025	Regulatory communication	1, 5	Removal of clozapine REMS
17	Stahl, 2021	Textbook	2	Subjective neuropsychiatric adverse effects
18	Cascade et al., 2009	Real-world data report	2	Common SSRI adverse effects
19	Hazell and Shakir, 2006	Systematic review	1	Magnitude of underreporting
20	FDA, 2004	Regulatory communication	2, 5	2004 boxed warning directive
21	Hammad et al., 2006	Pooled analysis of RCTs	2	Attribution of suicidality
22	Harpaz et al., 2012	Methodological review	3	Machine learning for signal detection
23	Harpaz et al., 2014	Narrative review	3	Text mining for adverse events
24	Golder et al., 2025	Scoping review	3, 4	NLP for adverse events in EHRs
25	Baethge et al., 2019	Methodological guidance	Methods	Narrative review reporting standard
26	Green et al., 2006	Methodological guidance	Methods	Narrative review methods
27	Moore et al., 2007	Analysis of FAERS reports	1	Growth in report volume
28	Lopez-Gonzalez et al., 2009	Systematic review	1	Determinants of underreporting
29	Corrigan et al., 2014	Narrative review	2	Stigma and care seeking
30	Thornicroft et al., 2009	Cross-sectional survey	2	Discrimination in mental illness
31	Montejo et al., 2018	Narrative review	2	Under-recognition of sexual dysfunction
32	Shefer et al., 2014	Qualitative study	2	Diagnostic overshadowing
33	Sakaeda et al., 2013	Methodological study (FAERS)	1	Disproportionality analysis of FAERS
34	Bate and Evans, 2009	Methodological review	1	Disproportionality measures
35	Almenoff et al., 2007	Methodological review	1	Bayesian signal detection
36	Alvarez et al., 2010	Retrospective validation study	1	Performance of statistical signal detection
37	Pariente et al., 2007	Pharmacoepidemiological study	1	Notoriety bias
38	Hoffman et al., 2014	Analysis of FAERS reports	1	Weber effect in modern FAERS
39	Waddell and Taylor, 2008	Instrument development study	2	Patient-rated adverse effect measures
40	Stone et al., 2009	Pooled analysis of RCTs	2	Age-dependence of suicidality risk
41	Friedman and Leon, 2007	Perspective	2, 5	Extension of boxed warning
42	Carbon et al., 2017	Meta-analysis	2	Tardive dyskinesia prevalence
43	De Hert et al., 2012	Narrative review	2	Antipsychotic metabolic effects
44	ADA/APA/AACE/NAASO, 2004	Consensus statement	2	Metabolic monitoring recommendations
45	Solmi et al., 2018	Systematic review and meta-analysis	2	Treatment of tardive dyskinesia
46	Mojtabai and Olfson, 2010	National survey analysis	2	Trends in psychotropic polypharmacy
47	Kukreja et al., 2013	Narrative review	2	Polypharmacy in severe illness
48	Masnoon et al., 2017	Systematic review	2	Definitions of polypharmacy
49	Zimmerman et al., 2002	Cross-sectional study	2	Trial eligibility of routine patients
50	Kennedy-Martin et al., 2015	Literature review	2	Representativeness of trial samples
51	Konrat et al., 2012	Methodological review of RCTs	2	Underrepresentation of older adults
52	Bhatnagar et al., 2022	Regulatory perspective	2, 5	Exclusion of pregnant and lactating individuals
53	Downing et al., 2017	Cohort study	1	Post-marketing safety events
54	Robb et al., 2012	Perspective (regulatory)	1	Strengths and limits of Sentinel
55	Sentinel Operations Center/FDA, 2025	Regulatory assessment report	1, 5	Current Sentinel priorities
56	Sarker et al., 2015	Systematic review	3	Social media pharmacovigilance
57	Nikfarjam et al., 2015	Methods development study	3	NLP on social media
58	Weissenbacher et al., 2019	Shared-task overview	3	Benchmark social media text mining
59	Rieke et al., 2020	Narrative review	3, 5	Privacy-preserving data integration
60	Iqbal et al., 2017	Methods development and validation study	3, 4	NLP in psychiatric records
61	Cai et al., 2024	Methods evaluation study	3, 4	LLM extraction of adverse events
62	Sun et al., 2024	Methods evaluation study	3, 4	LLM performance on extraction
63	Zitu et al., 2025	Narrative review	3, 4	Limitations of LLMs
64	Dimitsaki et al., 2024	Scoping review	3, 4	Maturity of AI evidence
65	Choi et al., 2016	Methods development study	3	Longitudinal EHR modeling
66	Miotto et al., 2016	Methods development study	3	Longitudinal EHR modeling
67	Torous et al., 2016	Platform development study	3	Digital phenotyping
68	Insel, 2017	Perspective	3	Digital phenotyping
69	Topol, 2019	Narrative review	4, 5	Limits of medical AI
70	Rajkomar et al., 2019	Narrative review	4, 5	Limits of medical AI
71	Obermeyer et al., 2019	Cross-sectional algorithm audit	5	Algorithmic bias
72	Chen et al., 2021	Narrative review	5	Equity in machine learning
73	Ball and Dal Pan, 2022	Regulatory perspective	4, 5	Human oversight of AI in pharmacovigilance
74	Bate and Hobbiger, 2021	Narrative review	4, 5	Barriers to AI adoption
75	FDA, 2025	Regulatory draft guidance	5	Credibility framework for AI

## References

[1] (2026). Results from the 2021 National Survey on Drug Use and Health (NSDUH): key substance use and mental health indicators in the United States. https://library.samhsa.gov/product/results-2021-national-survey-drug-use-and-health-nsduh-key-substance-use-and-mental-health.

[2] Kessler RC, Petukhova M, Sampson NA, Zaslavsky AM, Wittchen HU (2012). Twelve-month and lifetime prevalence and lifetime morbid risk of anxiety and mood disorders in the United States. Int J Methods Psychiatr Res.

[3] Fulton JJ, Calhoun PS, Wagner HR (2015). The prevalence of posttraumatic stress disorder in Operation Enduring Freedom/Operation Iraqi Freedom (OEF/OIF) Veterans: a meta-analysis. J Anxiety Disord.

[4] Greenberg PE, Fournier AA, Sisitsky T, Simes M, Berman R, Koenigsberg SH, Kessler RC (2021). The economic burden of adults with major depressive disorder in the United States (2010 and 2018). Pharmacoeconomics.

[5] Krystal JH, Abdallah CG, Sanacora G, Charney DS, Duman RS (2019). Ketamine: a paradigm shift for depression research and treatment. Neuron.

[6] Carhart-Harris R, Giribaldi B, Watts R (2021). Trial of psilocybin versus escitalopram for depression. N Engl J Med.

[7] (2026). The importance of pharmacovigilance: safety monitoring of medicinal products. https://apps.who.int/iris/handle/10665/42493.

[8] (2026). Postmarket drug safety information for patients and providers. https://www.fda.gov/drugs/surveillance/postmarket-drug-safety-information-patients-and-providers.

[9] (2026). Adverse Event Reporting System (FAERS) quarterly data extract files. https://fis.fda.gov/extensions/FPD-QDE-FAERS/FPD-QDE-FAERS.html.

[10] Behrman RE, Benner JS, Brown JS, McClellan M, Woodcock J, Platt R (2011). Developing the Sentinel system - a national resource for evidence development. N Engl J Med.

[11] Platt R, Brown JS, Robb M, McClellan M, Ball R, Nguyen MD, Sherman RE (2018). The FDA Sentinel initiative - an evolving national resource. N Engl J Med.

[12] (2026). Postmarketing studies and clinical trials - implementation of section 505(o)(3) of the Federal Food, Drug, and Cosmetic Act. https://www.fda.gov/regulatory-information/search-fda-guidance-documents/postmarketing-studies-and-clinical-trials-implementation-section-505o3-federal-food-drug-and-cosmetic.

[13] (2026). Risk evaluation and mitigation strategies (REMS). https://www.fda.gov/drugs/drug-safety-and-availability/risk-evaluation-and-mitigation-strategies-rems.

[14] Gastaldon C, Raschi E, Kane JM, Barbui C, Schoretsanitis G (2021). Post-marketing safety concerns with esketamine: a disproportionality analysis of spontaneous reports submitted to the FDA Adverse Event Reporting System. Psychother Psychosom.

[15] (2026). Supplement approval letter, NDA 211243/S-029: SPRAVATO (esketamine) nasal spray, REMS modification. https://www.accessdata.fda.gov/drugsatfda_docs/appletter/2026/211243Orig1s029ltr.pdf.

[16] (2026). FDA removes risk evaluation and mitigation strategy (REMS) program for the antipsychotic drug clozapine. https://www.fda.gov/drugs/drug-safety-and-availability/fda-removes-risk-evaluation-and-mitigation-strategy-rems-program-antipsychotic-drug-clozapine.

[17] Andrade C (2010). Stahl’s essential psychopharmacology: neuroscientific basis and practical applications. Mens Sana Monogr.

[18] Cascade E, Kalali AH, Kennedy SH (2009). Real-world data on SSRI antidepressant side effects. Psychiatry (Edgmont).

[19] Hazell L, Shakir SA (2006). Under-reporting of adverse drug reactions: a systematic review. Drug Saf.

[20] (2026). Suicidality in children and adolescents being treated with antidepressant medications. https://www.fda.gov/Drugs/DrugSafety/PostmarketDrugSafetyInformationforPatientsandProviders/ucm161679.htm.

[21] Hammad TA, Laughren T, Racoosin J (2006). Suicidality in pediatric patients treated with antidepressant drugs. Arch Gen Psychiatry.

[22] Harpaz R, DuMouchel W, Shah NH, Madigan D, Ryan P, Friedman C (2012). Novel data-mining methodologies for adverse drug event discovery and analysis. Clin Pharmacol Ther.

[23] Harpaz R, Callahan A, Tamang S (2014). Text mining for adverse drug events: the promise, challenges, and state of the art. Drug Saf.

[24] Golder S, Xu D, O'Connor K, Wang Y, Batra M, Hernandez GG (2025). Leveraging natural language processing and machine learning methods for adverse drug event detection in electronic health/medical records: a scoping review. Drug Saf.

[25] Baethge C, Goldbeck-Wood S, Mertens S (2019). SANRA - a scale for the quality assessment of narrative review articles. Res Integr Peer Rev.

[26] Green BN, Johnson CD, Adams A (2006). Writing narrative literature reviews for peer-reviewed journals: secrets of the trade. J Chiropr Med.

[27] Moore TJ, Cohen MR, Furberg CD (2007). Serious adverse drug events reported to the Food and Drug Administration, 1998-2005. Arch Intern Med.

[28] Lopez-Gonzalez E, Herdeiro MT, Figueiras A (2009). Determinants of under-reporting of adverse drug reactions: a systematic review. Drug Saf.

[29] Corrigan PW, Druss BG, Perlick DA (2014). The impact of mental illness stigma on seeking and participating in mental health care. Psychol Sci Public Interest.

[30] Thornicroft G, Brohan E, Rose D, Sartorius N, Leese M (2009). Global pattern of experienced and anticipated discrimination against people with schizophrenia: a cross-sectional survey. Lancet.

[31] Montejo AL, Montejo L, Baldwin DS (2018). The impact of severe mental disorders and psychotropic medications on sexual health and its implications for clinical management. World Psychiatry.

[32] Shefer G, Henderson C, Howard LM, Murray J, Thornicroft G (2014). Diagnostic overshadowing and other challenges involved in the diagnostic process of patients with mental illness who present in emergency departments with physical symptoms - a qualitative study. PLoS One.

[33] Sakaeda T, Tamon A, Kadoyama K, Okuno Y (2013). Data mining of the public version of the FDA Adverse Event Reporting System. Int J Med Sci.

[34] Bate A, Evans SJ (2009). Quantitative signal detection using spontaneous ADR reporting. Pharmacoepidemiol Drug Saf.

[35] Almenoff JS, Pattishall EN, Gibbs TG, DuMouchel W, Evans SJ, Yuen N (2007). Novel statistical tools for monitoring the safety of marketed drugs. Clin Pharmacol Ther.

[36] Alvarez Y, Hidalgo A, Maignen F, Slattery J (2010). Validation of statistical signal detection procedures in eudravigilance post-authorization data: a retrospective evaluation of the potential for earlier signalling. Drug Saf.

[37] Pariente A, Gregoire F, Fourrier-Reglat A, Haramburu F, Moore N (2007). Impact of safety alerts on measures of disproportionality in spontaneous reporting databases: the notoriety bias. Drug Saf.

[38] Hoffman KB, Dimbil M, Erdman CB, Tatonetti NP, Overstreet BM (2014). The Weber effect and the United States Food and Drug Administration's Adverse Event Reporting System (FAERS): analysis of sixty-two drugs approved from 2006 to 2010. Drug Saf.

[39] Waddell L, Taylor M (2008). A new self-rating scale for detecting atypical or second-generation antipsychotic side effects. J Psychopharmacol.

[40] Stone M, Laughren T, Jones ML (2009). Risk of suicidality in clinical trials of antidepressants in adults: analysis of proprietary data submitted to US Food and Drug Administration. BMJ.

[41] Friedman RA, Leon AC (2007). Expanding the black box - depression, antidepressants, and the risk of suicide. N Engl J Med.

[42] Carbon M, Hsieh CH, Kane JM, Correll CU (2017). Tardive dyskinesia prevalence in the period of second-generation antipsychotic use: a meta-analysis. J Clin Psychiatry.

[43] De Hert M, Detraux J, van Winkel R, Yu W, Correll CU (2011). Metabolic and cardiovascular adverse effects associated with antipsychotic drugs. Nat Rev Endocrinol.

[44] American Diabetes Association, American Psychiatric Association, American Association of Clinical Endocrinologists, North American Association for the Study of Obesity (2004). Consensus development conference on antipsychotic drugs and obesity and diabetes. Diabetes Care.

[45] Solmi M, Pigato G, Kane JM, Correll CU (2018). Treatment of tardive dyskinesia with VMAT-2 inhibitors: a systematic review and meta-analysis of randomized controlled trials. Drug Des Devel Ther.

[46] Mojtabai R, Olfson M (2010). National trends in psychotropic medication polypharmacy in office-based psychiatry. Arch Gen Psychiatry.

[47] Kukreja S, Kalra G, Shah N, Shrivastava A (2013). Polypharmacy in psychiatry: a review. Mens Sana Monogr.

[48] Masnoon N, Shakib S, Kalisch-Ellett L, Caughey GE (2017). What is polypharmacy? A systematic review of definitions. BMC Geriatr.

[49] Zimmerman M, Mattia JI, Posternak MA (2002). Are subjects in pharmacological treatment trials of depression representative of patients in routine clinical practice?. Am J Psychiatry.

[50] Kennedy-Martin T, Curtis S, Faries D, Robinson S, Johnston J (2015). A literature review on the representativeness of randomized controlled trial samples and implications for the external validity of trial results. Trials.

[51] Konrat C, Boutron I, Trinquart L, Auleley GR, Ricordeau P, Ravaud P (2012). Underrepresentation of elderly people in randomised controlled trials. The example of trials of 4 widely prescribed drugs. PLoS One.

[52] Bhatnagar S, Bello CL, Pang L, Mugundu G (2022). Considerations for pregnant and lactating women in clinical drug development: a regulatory perspective. Clin Pharmacol Ther.

[53] Downing NS, Shah ND, Aminawung JA, Pease AM, Zeitoun JD, Krumholz HM, Ross JS (2017). Postmarket safety events among novel therapeutics approved by the US Food and Drug Administration between 2001 and 2010. JAMA.

[54] Robb MA, Racoosin JA, Sherman RE (2012). The US Food and Drug Administration's Sentinel Initiative: expanding the horizons of medical product safety. Pharmacoepidemiol Drug Saf.

[55] (2026). Sentinel Operations Center, U.S. Food and Drug Administration: PDUFA VII commitment: an assessment of the Sentinel System (2022 to 2024). https://www.fda.gov/industry/prescription-drug-user-fee-amendments/pdufa-vii-commitment-assessment-sentinel-system-2022-2024.

[56] Sarker A, Ginn R, Nikfarjam A (2015). Utilizing social media data for pharmacovigilance: a review. J Biomed Inform.

[57] Nikfarjam A, Sarker A, O'Connor K, Ginn R, Gonzalez G (2015). Pharmacovigilance from social media: mining adverse drug reaction mentions using sequence labeling with word embedding cluster features. J Am Med Inform Assoc.

[58] Weissenbacher D, Sarker A, Magge A (2019). Overview of the Fourth Social Media Mining for Health (SMM4H) shared tasks at ACL 2019. Proceedings of the Fourth Social Media Mining for Health Applications Workshop & Shared Task.

[59] Rieke N, Hancox J, Li W (2020). The future of digital health with federated learning. NPJ Digit Med.

[60] Iqbal E, Mallah R, Rhodes D (2017). ADEPt, a semantically-enriched pipeline for extracting adverse drug events from free-text electronic health records. PLoS One.

[61] Li Y, Li J, He J, Tao C (2024). using large language models to extract adverse events from surveillance reports - a use case with influenza vaccine adverse events. PLoS One.

[62] Sun Z, Pergola G, Wallace BC, He Y (2024). Leveraging ChatGPT in pharmacovigilance event extraction: an empirical study. Proceedings of the 18th Conference of the European Chapter of the Association for Computational Linguistics.

[63] Zitu MM, Owen D, Manne A, Wei P, Li L (2025). Large language models for adverse drug events: a clinical perspective. J Clin Med.

[64] Dimitsaki S, Natsiavas P, Jaulent MC (2024). Applying AI to structured real-world data for pharmacovigilance purposes: scoping review. J Med Internet Res.

[65] Choi E, Bahadori MT, Sun J, Kulas J, Schuetz A, Stewart W (2016). RETAIN: an interpretable predictive model for healthcare using reverse time attention mechanism. Advances in Neural Information Processing Systems.

[66] Miotto R, Li L, Kidd BA, Dudley JT (2016). Deep patient: an unsupervised representation to predict the future of patients from the electronic health records. Sci Rep.

[67] Torous J, Kiang MV, Lorme J, Onnela JP (2016). New tools for new research in psychiatry: a scalable and customizable platform to empower data driven smartphone research. JMIR Ment Health.

[68] Insel TR (2017). Digital phenotyping: technology for a new science of behavior. JAMA.

[69] Topol EJ (2019). High-performance medicine: the convergence of human and artificial intelligence. Nat Med.

[70] Rajkomar A, Dean J, Kohane I (2019). Machine learning in medicine. N Engl J Med.

[71] Obermeyer Z, Powers B, Vogeli C, Mullainathan S (2019). Dissecting racial bias in an algorithm used to manage the health of populations. Science.

[72] Chen IY, Pierson E, Rose S, Joshi S, Ferryman K, Ghassemi M (2021). Ethical machine learning in healthcare. Annu Rev Biomed Data Sci.

[73] Ball R, Dal Pan G (2022). "Artificial intelligence" for pharmacovigilance: ready for prime time?. Drug Saf.

[74] Bate A, Hobbiger SF (2021). Artificial intelligence, real-world automation and the safety of medicines. Drug Saf.

[75] (2026). Considerations for the use of artificial intelligence to support regulatory decision-making for drug and biological products. https://www.fda.gov/regulatory-information/search-fda-guidance-documents/considerations-use-artificial-intelligence-support-regulatory-decision-making-drug-and-biological.

